# Multidrug-resistant and enterotoxigenic methicillin-resistant Staphylococcus aureus isolated from raw milk of cows at small-scale production units

**DOI:** 10.5455/javar.2022.i575

**Published:** 2022-03-10

**Authors:** Hamza Mohamed Eid, Heba Sayed El-Mahallawy, Sahar Roshdi Mohammed, Nehal Ezzat Yousef Mohammed, Nada Hussein Eidaroos

**Affiliations:** 1 Department of Bacteriology, Immunology and Mycology, Faculty of Veterinary Medicine, Suez Canal University, Ismailia, Egypt; 2 Department of Animal Hygiene, Zoonoses, and Animal Behaviour and Management, Faculty of Veterinary Medicine, Suez Canal University, Ismailia, Egypt; 3 Head Research of Bacteriology and Chief of Bacteriology Department, Animal Health Research Institute, Dokki, Egypt; 4 Food Inspector, Animal Health Research Institute, Damietta, Egypt

**Keywords:** Antimicrobial resistance, cow’s milk, enterotoxin genes, MRSA, *Staphylococcus aureus*, virulence genes

## Abstract

**Objective:**

*Staphylococcus aureus *(*S. aureus*) has evolved as one of the most significant bacteria causing food poisoning outbreaks worldwide. This study was carried out to investigate the prevalence, antibiotic sensitivity, virulence, and enterotoxin production of *S. aureus* in raw milk of cow from small-scale production units and house-raised animals in Damietta governorate, Egypt.

**Material and Methods:**

The samples were examined bacteriologically, and antimicrobial sensitivity testing was carried out. Moreover, isolates were characterized by the molecular detection of antimicrobial resistance, virulence, and enterotoxin genes.

**Results:**

Out of 300 milk samples examined, *S. aureus* was isolated from 50 samples (16.7%). Antibiotic sensitivity testing revealed that isolates were resistant to β-lactams (32%), tetracycline (16%), and norfloxacin (16%); however, they showed considerable sensitivity to ceftaroline and amikacin (72%). Multidrug-resistance (MDR) has been observed in eight isolates (16%), with a MDR index (0.5) in all of them. Of the total *S. aureus* isolates obtained, methicillin-resistant *S. aureus* (MRSA) has been confirmed molecularly in 16/50 (32%) and was found to carry *mecA* and *coa* genes, while virulence genes; *hlg* (11/16, 68.75%) and *tsst* (6/16, 37.5%) were amplified at a lower percentage, and they showed a significant moderate negative correlation (*r* = −0.59, *p*-value > 0.05). Antibiotic resistance genes have been detected in resistant isolates relevant to their phenotypic resistance: *blaZ* (100%), *tetK *(50%), and *norA* (50%). Fifty percent of MRSA isolates carried the *seb* enterotoxin gene.

**Conclusion:**

High detection rate of MRSA and MDR isolates from milk necessitates the prompt implementation of efficient antimicrobial stewardship guidelines, especially at neglected small-scale production units.

## Introduction

*Staphylococcus aureus* (*S. aureus*) is considered one of the most significant pathogens in recent decades. It is ranked as the third causative agent of foodborne illnesses globally, drawing the attention of public health programs worldwide [1]. Staphylococcal foodborne poisoning (SFP) results from the consumption of contaminated foods like meat, milk, or eggs (mainly animal products) by *S. aureus* enterotoxins (SEs), which are heat-resistant proteins that have high stability in the digestive tract [2,3]. SFP caused by enterotoxigenic *S. aureus* grows quickly and can be very dangerous to humans [3]. 

There are around 20 distinct SEs that are encoded by genes on various staphylococcal pathogenicity islands [4], with the most common classical SEs being *sea*, *seb*, *sec*, *sed*, and *see*, accounting for 95% of SFP cases. The remaining 5% of infections are caused by recently identified SEs [5]. SMEs are a subgroup of the staphylococcal superantigen family, including enterotoxin-like serotypes and toxic shock syndrome (TSS) toxin 1 (TSST-1). These superantigen toxins are potent nonspecific stimulators of T cells that link the MHC II receptors on antigen-presenting cells and the Vβ chains on T-cell receptors. This leads to bypassing the normal antigen-specific restrictions of immune cells, resulting in rapid T-cell expansion and massive release of the proinflammatory cytokine, which can kill patients [6,7]. SEB is one of the most virulent superantigens of these enterotoxins and is designated as a class B bioweapon [8]. Food and Drug Administration-approved antibiotics for the treatment of *S. aureus* infection in humans and animals are divided into two classes: β-lactams (e.g., amoxicillin, penicillin, oxacillin, ceftiofur, hetacillin, and cephapirin) and lincosamides (e.g., pirlimycin) [9]. Antibiotics are used indiscriminately and in large quantities in animal and human medicine, resulting in the emergence of multidrug-resistant (MDR) bacteria, like methicillin-resistant *S. aureus* (MRSA). The pathogenesis of *S. aureus* is aggravated by the acquisition of resistance to several antibiotics, which causes a challenge in the treatment of *S. aureus* infection [10]. The World Health Organization has listed MRSA, often known as “superbug” or “resistant staph,” as a high-priority organism for more research and treatment [11]. The emergence of livestock-associated MRSA (from livestock and their products) is alarming due to its increasing rate and the high risk of zoonotic transmission worldwide.These strains are to blame for therapeutic failures, limiting the options for treating serious infections [12].

Consumption of raw milk has gradually increasedin tandemwith consumer interest in minimally processed foods. However, milk can be a vehicle for zoonotic pathogens from animals to humans. *S. aureus* is the common agent of clinical or subclinical mastitis in animals, and drinking raw milk poses a great risk to consumer health [13]. 

Due to raw milk contamination by pathogenic bacteria, notably enterotoxigenic *S. aureus*, and the severe increase in antimicrobial resistance, research on the epidemiological features of *S. aureus* in raw milk has received global attention.The phenotypic and genotypic background and antibiotic resistance of *S. aureus*, particularly MRSA, isolated from raw milk, are regularly monitored. The presence of small-scale production units and house-raised animals represents a major challenge in any community. Although they add to the economy through their units’ production, they are skipped from national monitoring programs that usually include large companies; thus, they represent a great threat to human health if their animals or animal products are contaminated with life-threatening agents. Therefore, this study was carried out to know to what extent virulent and enterotoxigenic-resistant *S. aureus* strains are present in the raw milk of cows in these units in the study area.

## Materials and Methods

### Ethical approval

The present study’s sample collection and processing have been reviewed and approved by the Scientific Research Committee and Bioethics Board of Suez Canal University, Faculty of Veterinary Medicine, Ismailia, Egypt [No. 2021042].

### Sample size calculations

The sample size was calculated according to G*Power software version 3.1.9.6 [14–16]. A correlation analysis was proposed, and a minimum total sample size of 292 samples was sufficient to detect the effect size of 0.189 and a power of 0.95 at a significance probability level of 0.05 and partial *R*^2^ of 0.036, where *f* is the effect size = 0.189, *α* = 0.05, *β* = 0.05, power = 1-*β* = 0.95. According to the sample size calculations, 300 samples were used in the current study.

### Sample collection

A total of 300 milk samples were collected from apparently healthy cows by small-scale holders, individual household producers, and small farms in 10 regions of Damietta and New Damietta cities in Damietta, Egypt. Samples were collected under complete aseptic conditions after washing the udder with soap and water, disinfection with 70% ethyl alcohol, complete dryness, and discarding the foremilk. About 15 ml of milk was collected in clean, labeled plastic centrifuge tubes, transferred directly to the laboratory in an icebox, and processed within an hour of collection, after 3 min. After centrifugation at 3,000 rpm, the sediment was used for bacteriological examination.

### Isolation and identification of S. aureus

*S. aureus* identification was carried out as previously described [17]. Loopfuls from deposits of the centrifuged milk were pre-enriched in 10 ml of peptone water for 18–24 h at 37°C before being plated on the surface of the following media: nutrient agar, mannitol salt agar, Baird–Parker agar, and 5% sheep blood agar (Oxoid, Hampshire, UK). All plates were incubated at 37°C for 24–48 h. The plates were examined for colony characteristics, cellular morphology in Gram stain (Gram-positive, grapes-like cocci, and arranged in clusters), culture purity, hemolysis, and pigment production. The catalase test was carried out on grape-like Gram-positive bacteria, and positive isolates were examined using slide and tube coagulase tests to differentiate *S. aureus* from other *Staphylococcus* species.

### Antibiotic susceptibility testing

The sensitivity of the obtained isolates was assessed using the Kirby–Bauer disk diffusion method on Mueller–Hinton agar (Oxoid, Hampshire, UK), following the protocol of the Clinical and Laboratory Standard Institute for Antimicrobial Susceptibility Testing [18], against the most commonly used antimicrobials in humans and farm animals in the study area, namely penicillin (P) (10 µg),) oxacillin (OX) (1 µg), cefoxitin (FOX 30 µg), ceftaroline (Rx) (30 µg), amikacin (AK) (30 µg), gentamycin (CN) (10 µg), norfloxacin (NOR) (10 µg), oxytetracycline (OT) (30 µg), chloramphenicol (C) (30 µg), sulfa-trimethoprim (SXT) (25 µg) (Oxoid, Hampshire, UK). In brief, a suspension of each pure identified isolate was prepared in Mueller–Hinton broth (Oxoid, Hampshire, UK) until it matched the turbidity of 0.5 McFarland standard, and sterile cotton swabs were used to evenly streak Mueller–Hinton agar plates. After 3–5 min, antimicrobial disks were distributed evenly and firmly into the agar and incubated 37°C for 24–48 h in an inverted position. The zone of inhibition of growth was measured to evaluate the sensitivity, as suggested by the Clinical and Laboratory Standard Institute for Antimicrobial Susceptibility Testing [18]. 

### Determination of MDR and MAR index among S. aureus isolates

Multidrug-resistant bacteria (MDR) is a synonym designated for isolates found resistant to at least one agent in three or more antimicrobial classes [19]. The multiple antibiotic resistance (MAR) index was determined using the previously described formula: *a*/*b* [20], where *a* indicates the number of antibiotics to which the isolate exhibits resistance, while *b* represents the total number of the tested antimicrobials to which the isolate was exposed. Isolates with a MAR index exceeding 0.2 come from a high-risk contamination source that uses various antibiotics, whereas bacteria with a MAR index of less than 0.2 come from a source that uses fewer antibiotics. The MAR index of a fully resistant isolate is 1.0.

### Molecular characterization of MRSA isolates

DNA was extracted from pure overnight cultures according to the QIAamp DNA Mini Kit instructions (Qiagen, Germany, GmbH).All phenotypical oxacillin- and cefoxitin-resistant *S. aureus* isolates were screened for the presence of the *mecA* gene for molecular detection of MRSA isolates; the antibiotic resistance genes *blaZ* (penicillin), *norA* (norofloxacin), and *tetK* (tetracycline) genes (relevant to the observed phenotypic resistance); and *coa*, *tsst*-1, and *hlg* virulence genes according to previously described *polymerase chain reaction (*PCR) protocols (Table 1). Each reaction mixture consisted of 12.5 µl of Emerald Amp GT PCR master mix (2x premix) (Takara, Japan), 1 µl (20 pmol) of forward and reverse primers (Metabion, Germany), and 6 µl of template DNA, and completed to 25 µl with PCR grade water. In addition, a multiplex PCR was carried out to detect staphylococcal enterotoxin genes (*sea*, *seb*, *sec*, *sed*, and *see*) as previously mentioned (Table 1), where 1 µl (20 pmol) of forward and reverse primers and 6 µl of template DNA were added to 25 µl of PCR master mix in a 50 µl final reaction volume. Positive control strains supplied from the Department of Bacteriology, Animal Health Research Institute, Dokki, Egypt, were used in each run, and DNAse RNAse free water was used as a negative control. Amplification products were electrophoresed using a 1.5% agarose gel (Applichem, Germany, GmbH) dipped in 1 × TBE buffer using 0.5 μg/ml ethidium bromide against a 100-bp molecular ladder (Fermentas, Thermo, Germany), followed by photographing using a photo documentation system (Alpha Innotech, Biometra).

### Statistical analysis

Data were handled and statistically tested for normality in SPSS 20.0 (IBM-SPSS Inc., Chicago, IL) at the 0.05 level using the Shapiro–Wilk test. Nonparametric data analysis was used (Shapiro–Wilk < 0.05). A correlation matrix was performed to check the relationship between virulence, enterotoxin, and antimicrobial resistance genes using the “cor” function in R software version (3.6.1) and visualized using the “corrplot” package. Additionally, the “cor.mtest” function was used to evaluate the significance of the correlation (*p*-value = 0.05).

## Results

### Prevalence of S. aureus in raw milk samples

Out of 300 milk samples examined, *Staphylococcus* isolates were recovered from100 samples (33.33%), where *S. aureus* had been detected in 50 (16.7%) samples based on the morphological, cultural, and biochemical characteristics. 

### Antimicrobial sensitivity testing

Antimicrobial sensitivity testing showed that penicillin, oxacillin, and cefoxitin were the most resistant to antimicrobials (16/50, 32%), followed by oxytetracycline and norfloxacin (8/50, 16%). However, the isolates were very sensitive to most of the other antimicrobials that were used.

### Detection of MDR and MAR index for S. aureus isolates

Among 50 *S. aureus* isolates, 8 (16%) isolates were MDR, and their MAR index was ≥ 0.2 (0.5). All other isolates had a MAR index ≤ 0.2, and none had a MAR index ≥ 1.0.

**Table 1.** Oligonucleotide primers sequences for virulence, antibiotic resistance, and enterotoxin genes of *S. aureus*.

Target gene	Sequence (5′-3′)	Amplified product (bp)	Annealing temperature	Reference
*mecA*	GTAGAAATGACTGAACGTCCGATA A	310	50°C/30 sec	[21]
	CCAATTCCACATTGTTTCGGTCTAA			
*tsst*	ACCCCTGTTCCCTTATCATC	326	50°C/30 sec	[22]
	TTTTCAGTATTTGTAACGCC			
*coa*	ATAGAGATGCTGGTACAGG	Four different types of bands may be detected 350 430 570 630	55°C/40 sec	[23]
	GCTTCCGATTGTTCGATGC			
*hlg*	GCCAATCCGTTATTAGAAAATGC	937	55°C/40 sec	[24]
	CCATAGACGTAGCAACGGAT			
*norA*	TTCACCAAGCCATCAAAAAG	620	50°C/40 sec	[25]
	CTTGCCTTTCTCCAGCAATA			
*blaZ*	ACTTCAACACCTGCTGCTTTC	173	54°C/30 sec	[26]
	TGACCACTTTTATCAGCAACC			
*tetK*	GTAGCGACAATAGGTAATAGT	360	54°C/40 sec	
	GTAGTGACAATAAACCTCCTA			
*sea*	GGTTATCAATGTGCGGGTGG	102	57°C/40 sec	[22]
	CGGCACTTTTTTCTCTTCGG			
*seb*	GTATGGTGGTGTAACTGAGC	164		
	CCAAATAGTGACGAGTTAGG			
*sec*	AGATGAAGTAGTTGATGTGTATGG	451		
	CACACTTTTAGAATCAACCG			
*sed*	CCAATAATAGGAGAAAATAAAAG	278		
	ATTGGTATTTTTTTTCGTTC			
*see*	AGGTTTTTTCACAGGTCATCC	209		
	CTTTTTTTTCTTCGGTCAATC			

**Table 2.** Antibiotic sensitivity of *S. aureus* isolated from the raw milk of cows.

Antimicrobial class	Antimicrobial agents	Sensitive	Intermediate	Resistant
		No. (%)	No. (%)	No. (%)
β-lactams	Penicillin (P)	26 (52)	8 (16)	16 (32)
	Oxacillin (OX)	26 (52)	8 (16)	16 (32)
Cephems	Cefoxitin (FOX)	26 (52)	8 (16)	16 (32)
	Ceftaroline (Rx)	36 (72)	14 (28)	--
Aminoglycosides	Gentamycin (CN)	32 (64)	18 (36)	--
	Amikacin (AK)	36 (72)	14 (28)	--
Quinolones	Norfloxacin (NOR)	26 (52)	16 (32)	8 (16)
Tetracyclines	Oxytetracyclin (OT)	26 (52)	16 (32)	8 (16)
Sulfonamides combination	Sulfa + trimethoprim (SXT)	30 (60)	20 (40)	--
Phenicols	Chloramphenicol (C )	32 (64)	18 (36)	--

R = Resistant; I = Intermediate; S = Sensitive.

**Table 3.** Results of molecular characterization of MRSA isolated from the raw milk of cows.

Sample No.	*mecA*	Antibiotic resistance genes			Virulence genes			Enterotoxin genes				
*blaZ*	*norA*	*tetK*	*coa*	*tsst*	*hlg*	*seb*	*sea*	*sec*	*sed*	*see*
1	+	+	+	+	+	-	+	-	-	-	-	-
2	+	+	+	+	+	-	+	-	-	-	-	-
3	+	+	+	+	+	+	+	+	-	-	-	-
4	+	+	+	+	+	+	+	-	-	-	-	-
5	+	+	+	+	+	-	+	+	-	-	-	-
6	+	+	+	+	+	-	+	-	-	-	-	-
7	+	+	+	+	+	-	+	+	-	-	-	-
8	+	+	+	+	+	-	-	-	-	-	-	-
9	+	+	-	-	+	+	-	+	-	-	-	-
10	+	+	-	-	+	+	-	-	-	-	-	-
11	+	+	-	-	+	+	-	-	-	-	-	-
12	+	+	-	-	+	+	-	+	-	-	-	-
13	+	+	-	-	+	-	+	+	-	-	-	-
14	+	+	-	-	+	-	+	+	-	-	-	-
15	+	+	-	-	+	-	+	+	-	-	-	-
16	+	+	-	-	+	-	+	-	-	-	-	-
Total (%)	16 (100)	16 (100)	8 (50)	8 (50)	16 (100)	6 (37.5)	11 (68.75)	8 (50)	-	-	-	-

### Molecular characterization of MRSA isolates

**Figure 1.**
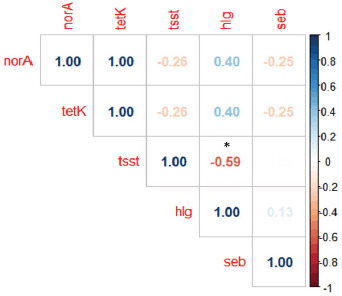
Correlation matrix between virulence, enterotoxin, and antibiotic resistance genes. Blue color indicates a positive correlation, while red color indicates a negative one. The degree of color intensity denotes the value of correlation. Variables were not significantly correlated, except *hlg* and *tsst* had a significant moderate negative correlation (*) (*p *> 0.05).

All phenotypically identified MRSA isolates (phenotypically resistant to oxacillin and cefoxitin) were confirmed by PCR by successfully amplifying both *coa* and* mecA* genes from all isolates. Furthermore, these isolates successfully amplified antibiotic resistance genes, *blaZ* (100%), *tetK *(50%), and *norA* (50%), in consistency with the results of the disk diffusion technique (Table 3). Of the 16 MRSA isolates obtained, virulence genes *hlg* (11/16, 68.75%) and *tsst* (6/16, 37.5%) were found. Only the *seb* gene has been detected in 50% (8/16) of MRSA isolates among the enterotoxin genes. An overview of the distribution of different genes in resistant and MDR isolates is shown in Table 4. According to Figure 1, there was a non-significant correlation for the presence of virulence, enterotoxin, and antimicrobial resistance genes, except between *hlg* and *tsst* there was a significant moderate negative correlation (*r* = −0.59, *p*-value > 0.05). The *seb* gene has a non-significant weak correlation with other virulence and antibiotic resistance genes (*r* = −0.25:0.13). The *mecA*, *blaZ*, and *coa* genes were not represented because they were present in all samples (100%) and had a zero standard deviation.

## Discussion

Foodborne outbreaks caused by milk and dairy products have resulted in hospitalizations and fatalities for people worldwide [27]. *Staphylococcus aureus* is a significant bacterium that causes toxin-mediated food poisoning. In this study, out of 300 raw milk samples examined, *S. aureus* (50, 16.7%) had been identified based on morphological, cultural, and biochemical characteristics. This is nearly similar to a recent study from Sharkia Governorate, Egypt (20%) [28]. Other studies conducted elsewhere, however, have recently reported a higher *S. aureus* prevalence; in China (43.1%) [13], Algeria (33.33%) [12], and Turkey (37.32%) [29]. Variations from country to country and even from region to region in the same country may be because of different sample sizes, antibiotics in animal husbandry, and hygiene standards for dairy cows.

Antibiotic sensitivity testing revealed that *S. aureus* isolates were resistant to penicillin and oxacillin and cefoxitin (32%), oxytetracycline (16%), and norfloxacin (16%), and antibiotic resistance genes, *blaZ* (32%)*, tetK *(16%)*,* and *norA *(16%),were detected in consistency with the phenotypic profile (Tables 3 and 4). Several studies, in recent years, have reported multiple resistances of *S. aureus* to a wide range of antibiotics, including β-lactams [12,13,30,31]. Consequently, β-lactam antibiotics are no longer effective in treating *S. aureus* infections. This may be due to the irresponsible use of antimicrobial agents at small milk-producing units away from veterinary supervision. Moreover, these units are usually skipped from the routine national veterinary monitoring programs.

Over the previous few decades, MRSA’s prevalence has expanded significantly and caused fatal infections [32].Overuse of β-lactam agents for treating mastitis in dairy cows and for prophylactic purposes may result in the emergence of MRSA in milk and, consequently, in dairy products made from it. In the current report, MDR isolates (at least resistant to one antimicrobial agent in three antibiotic classes) were observed in eight (16%) isolates, and MRSA was confirmed (16/50, 32%). Moreover, *mecA* and *coa* genes have been detected in all tested isolates that have been recognized phenotypically as MRSA. According to CLSI [18] guidelines, any *S. aureus* isolate found to be resistant to penicillinase-stable penicillins (e.g., oxacillin) or testing positive for the *mecA* gene should be reported as methicillin (oxacillin)-resistant *S. aureus* and considered resistant to other β-lactam agents. The MAR index is employed as a health risk assessment indicator to determine whether isolates come from high or low antibiotic usage contexts. The high-value MAR index detected in the MDR-resistant isolates from the present study (0.5) suggests that these isolates come from a source with high antibiotic usage and high selective pressure, which is typical at small-scale production units in the study area. In this regard, infected cows with MRSA are considered reservoirs that can transmit these resistant strains to other animals or humans, representing a significant threat to food safety and public health worldwide [33]. Previous reports detected a very high rate of MRSA from cases of mastitis in Turkey (90%) [34] and Brazil (23.3%) [35]. However, many others have reported MRSA from normally appearing milk: 2.5% [36] and 0.7% [37]in Italy;56.1% inUganda [38], and 2.29% in Algeria [12]. The consumption of antibiotic-resistant bacteria-contaminated food is becoming a serious hazard to global public health. Antibiotic resistance determinants in these pathogens can be transmitted to other clinically significant microorganisms. Recently, MRSA was reported to transfer methicillin resistance to human beings via milk and food [39,40]. Monitoring programs and quality assurance systems are essential for the dairy industry in order to keep *S. aureus*, MRSA, and other infections from spreading [41]. 

**Table 4.** Overview of phenotypic and genotypic profiles of resistant and MDR MRSA isolates.

Resistance pattern	No. of isolates	Phenotypic resistance profile	Antibiotic resistance genes	Virulence traits (No. of isolates)	Enterotoxin genes (No. of isolates)
MDR	8	P, OX, FOX, NOR, OX	*blaZ, norA, tetK*	*coa, tsst, hlg *(2)	*seb *(1)
				*coa, hlg* (5)	*seb *(2)
				*coa *(1)	*-*
Resistant	8	P, OX, FOX	*blaZ*	*coa, tsst *(4)	*seb *(2)
				*coa, hlg* (4)	*seb *(3)

Milk produced at small-scale production units is usually marketed to small villages. Due to bad habits and wrong traditions in these areas, milk is consumed raw and sometimes used to produce some homemade products without proper heat treatment. These homemade products are redistributed again to more consumers, which increases their implications if the milk is contaminated with bacteria of food safety concern. In this study, hemolysin *hlg* (68.75%) and TSS *tsst *(37.5%) genes were detected in MRSA isolates with a significant moderate negative correlation between them (Table 3, Fig. 1), which means they are inversely associated. To figure out how these strains might affect public health in the area where the study is taking place, scientists look for these genes.

Because of their ability to activate polyclonal T lymphocytes, enterotoxins are called superantigens because they suppress livestock immunity, leading to persistent intramammary infections [42]. Among the classical enterotoxins, the *sea* and *seb* enterotoxins are responsible for approximately 90% of staphylococcal food poisoning in humans globally [43], especially if they are generated prior to pasteurization of raw milk [44,45].In this study, eight (50%) isolates, out of all the tested MRSA strains, possessed the *seb* gene. The *seb* produced by MRSA is thought to be a primary cause of staphylococcal TSS [46]. This is of particular concern because, at small-scale production units, suitable storage facilities are scarce for milk until its distribution to customers in pre-urban areas, which allows favorable conditions for producing thermostable enterotoxins. This was nearly consistent with a recent study in Egypt where *the saw gene was detected in all (100%) S. aureus isolates from bovine milk, with similar occurrences of seb* and *sec* (33.3%) genes. However, none of the isolates carried *sea* or *sed* genes [47]. The problem is defined by the ability of these virulent strains to contaminate food and milk products and produce toxins without any apparent changes in the milk [36,48]. Further research is needed to determine how common *S. aureus* and MRSA are in the study area, how dangerous they are, and how many enterotoxins they make in homemade milk products.

## Conclusion

The findings in this study add to the available data concerning resistant *S. aureus* from the livestock community. Cows at small-scale production units in Damietta governorate are reservoirs for the virulent enterotoxigenic MRSA. These strains in milk are regarded as a possible health risk for food poisoning, especially if the milk is consumed after toxin production and before heat treatment. Public health education of the owners of the small production units about the careless and improper use of antimicrobials is critical. Frequent surveillance systems and programs should be employed to investigate the extent to which *Staphylococcus* spp. have become resistant.

## List of Abbreviations

MAR, Multiple antibiotic resistance index; MDR, Multidrug-resistant bacteria; MRSA, methicillin-resistant *S. aureus*; SEs, Staphylococcal enterotoxins;.
